# An innovative diagnostic strategy for the detection of rare molecular targets to select cancer patients for tumor-agnostic treatments

**DOI:** 10.18632/oncotarget.27343

**Published:** 2019-12-10

**Authors:** Antonio Marchetti, Alessia Di Lorito, Lara Felicioni, Fiamma Buttitta

**Affiliations:** ^1^ Laboratory of Diagnostic Molecular Oncology, Center for Advanced Studies and Technology (CAST), University of Chieti, Chieti, Italy; ^2^ Department of Medical and Oral Sciences and Biotechnologies, University of Chieti, Chieti, Italy; ^3^ Department of Pathology, SS Annunziata Clinical Hospital, Chieti, Italy

**Keywords:** tumor-agnostic treatments, microsatellite instability (MSI), neurotrophic receptor tyrosine kinase (NRTK), tissue microarrays (TMAs), tissue slice arrays (TSAs)

## Abstract

Targeted therapies are playing an increasing role in oncology. Among them, particular attention is nowadays reserved to histology-agnostic treatments. Rare molecular alterations affecting different neoplastic forms, such as Microsatellite Instability (MSI), Neurotropic Tyrosine Receptor Kinase (NTRK) gene fusions, etc., can allow efficient treatments, irrespective of the histologic type. Developing an effective testing strategy for the detection of rare molecular alterations is challenging.

We report an innovative diagnostic strategy for a rapid and economically affordable detection of this uncommon targets. Malignant tumor samples are selected at the time of histopathological diagnosis and further processed for simultaneous analysis of multiple samples on Tissue Micro Arrays (TMAs) and Tissue Slice Arrays (TSAs). The TSA approach was specifically designed for large scale screening of small biopsies. TMA sections and TSA were first screened by immunohistochemistry (IHC) for the expression of mismatch repair and TRK proteins. Positive cases were subjected to confirmation tests (fragment analysis/FISH/NGS).

In a series of 1865 malignant tumors, 48 (2.6%) MSI cases and 6 (0.3%) NTRK fusion cases were detected in 9 and 4 different tumor forms, respectively. On average, the TMA/TSA screening approach enabled IHC analysis of about 20 patients simultaneously with significant saving of time and costs. In addition, we have shown that multiplex IHC can further increment the throughput. A detailed procedure for application of this diagnostic approach in clinical practice is reported.

The strategy described may allow an efficient and sustainable selection of tumors carrying rare molecular targets, not to leave behind patients for effective agnostic treatments.

## INTRODUCTION

Target therapy has revolutionized the oncological approach to cancer patients carrying druggable molecular alterations [1]. New generation targeted therapies are increasingly effective and with fewer side effects, so no patient should be left behind without an appropriated treatment. A correct target treatment implies the identification of specific biomarkers alterations, as reported in dedicated guidelines [2–4].

Some of the biomarkers for target therapy are frequent events in particular tumor types (eg *RAS* mutations in colorectal cancer or *BRAF* mutations in melanoma). In these cases, accurate identification of the molecular alteration with dedicated methods is feasible and the cost and time of analysis to find a positive patient is acceptable [3, 4].

**Figure 1:**
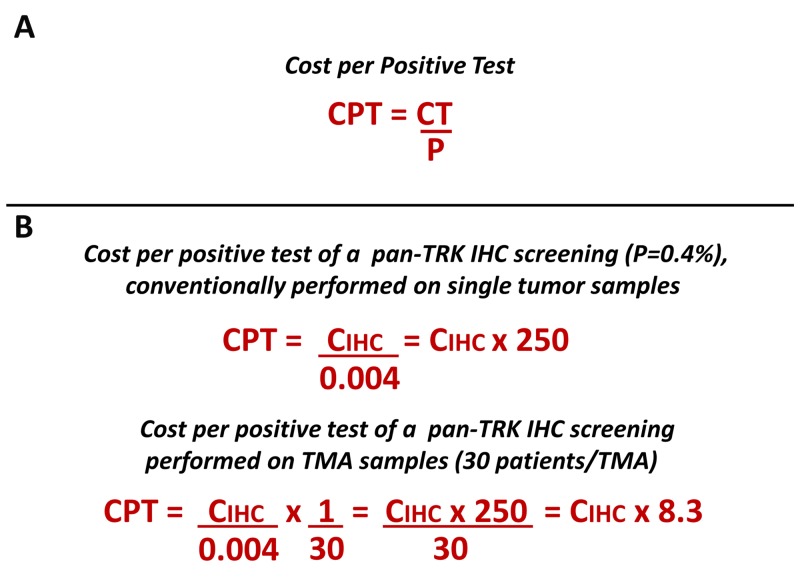
**(A)** The figure reports the equation to calculate the cost per positive test. CPT, cost per positive test; CT, cost per single test; P, prevalence of biomarker alteration. **(B)** The equation has been applied to calculate the cost of the pan-TRK IHC assay (CIHC) as an example of a test for the detection of a rare mutation (see the results section for furter details).

On the other hand, several biomarkers are rare or extremely rare events, while remaining valid to select cancer patients for very effective treatments. In practical terms, rare alterations may be defined as those present in less than 5% of patients. Within these alterations, important examples are *ALK1* and *ROS1* fusions, present in 3-5% and 1-2% of lung tumors, respectively, as well as in many other tumor types at lower prevalence rates [5–7]. The detection of rare mutations with a mono-marker test implies long time frames and high costs to identify a positive/druggable patient. Consider that the cost per positive test (CPT) is inversely related to the prevalence of the genomic alteration, as reported in the equation in Figure 1A.

Moreover, a mass of data produced by next generation sequencing in the last years indicate that some biomarkers are no longer restricted to specific tumor types, leading to histology agnostic treatments [8, 9]. This new therapeutic vision requires the analysis of molecular targets in many different tumor types if not in all, as in the case of the rare alterations affecting *mismatch repair* and *NTRK* genes [10, 11].

Two approaches are possible to meet these new diagnostic needs: 1) screening with methods applicable on a large scale as IHC followed by orthogonal tests (FISH, RT-PCR, Next generation sequencing) to confirm the alterations identified; 2) a direct and extended approach to all tumors through massive parallel sequencing. However, even a simple screening test, if extended to all currently needed biomarkers in clinical practice, and to all neoplastic forms, is not practical as it would have unacceptable timing and costs.

On the other hand, a large-scale NGS approach with large gene panels is desirable but at the moment, the costs and the low diffusion of the technology make it not realistically feasible [12].

Driven by these management difficulties, we have developed a diagnostic strategy based on large scale IHC screening of rare molecular alterations on tissue microarrays (TMAs) and Tissue Slice Arrays (TSAs) (see further text) to select cancer patients for histology-agnostic therapies. The approach has been finely tuned in order to meet the diagnostic needs of a standard pathology laboratory.

## RESULTS

A diagnostic strategy for the detection of rare molecular targets to select cancer patients for histology-agnostic treatments has been devised, as described in detail in the Material and Methods section. The innovative workflow provides that malignant tumor samples are identified by histological examination and subdivided into large and small samples based on size, regardless of the type of malignancy, for further processing. Very small samples, with inadequate amount of tissue for molecular diagnosis are excluded.

In a retrospective series, corresponding to a standard six months routine activity in our department (Table 1), malignant tumors (total n. 1865) accounted for about 15% of cases submitted for histopathological diagnosis and 311 malignant samples were collected each month, of which 146 (47%) were large samples, having at least one fragment above 0.5 cm^2^, and the remaining 165 (53%) were small samples.

**Table 1:** Retrospective series of different malignant tumors divided according to the size of embedded samples

Malignant Tumor	Samples		Total
	small	large	
**Bladder**	111	18	**129**
**Prostate**	87	90	**177**
**Kidney**	0	27	**27**
**Testis**	0	9	**9**
**Penis**	3	0	**3**
**Gynecopathological**	87	114	**201**
**Gastrointestinal and hepatic**	78	81	**159**
**Pancreas**	3	15	**18**
**Lung**	77	55	**132**
**Thyroid**	0	22	**22**
**Salivary**	0	10	**10**
**Lymph Node**	18	60	**78**
**Oral cavity**	27	9	**36**
**Breast**	345	366	**711**
**Skin**	153	0	**153**
**Total (%)**	989 (53)	876 (47)	**1865 (100)**
**Total per month**	165	146	**311**

Large samples were selected for the construction of TMA blocks using 2 (2mm) cores per tumor. The reliability of IHC staining for the expression of driver mutations performed on 2 (2mm) TMA cores versus the original tissue sections was investigated on a series of 60 selected blocks from lung neoplastic lesions including 23 blocks carrying *ALK* gene fusion, 12 with *ROS1* gene fusion, 3 with *NTRK1* gene fusion, and a series of 12 blocks from selected colorectal carcinomas with absence of mismatch repair protein expression in 7 cases. Two (2 mm) cores were representative of the tumor immunophenotypic profiling in 100% of cases with concordant data in the two cores of each patient (data not shown).

**Figure 2:**
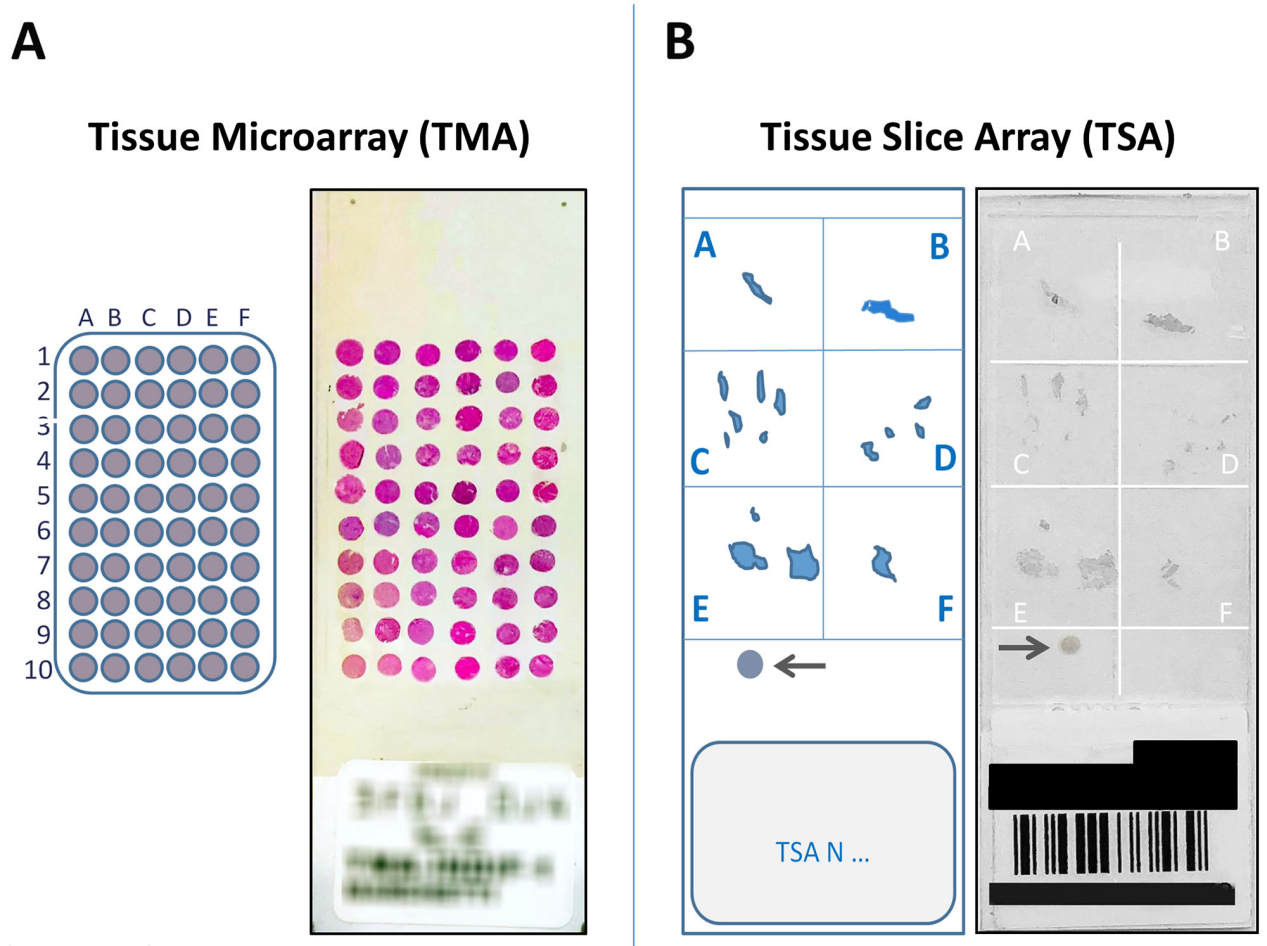
**(A)** The figure shows a 5 μm section from a 60 cores (30 tumors) Tissue MicroArray (TMA), obtained from large samples, stained with Hematoxylin–eosin, corresponding scheme on the left. **(B)** An example of a Tissue Slice Array (TSA), obtained from small samples, immunostained with the VENTANA pan-TRK (EPR17341) assay, corresponding scheme on the left. The arrows indicate an internal positive control.

It is advisable not to process tissues samples below 0.5 cm^2^ for TMA construction due to the paucity of biological material available. In these cases, in order to reduce the time and costs for the screening of rare molecular targets, we decided to proceed with the preparation of TSAs, an innovative approach that allows to maintain the integrity of tissue blocks, while ensuring the possibility of simultaneously examining multiple samples (Figure 2), as detailed in Materials and Methods.

Five micrometers TMA sections and TSAs were screened by IHC to evaluate mismatch repair and TRK A, B, C proteins expression. In case of neoplastic forms for which routine clinical molecular testing is planned (i.e non- small cell lung cancer, colorectal carcinoma, melanoma etc.), the search for rare biomarker alterations was conducted after the analysis of conventional predictive tests. Positive cases were subjected to confirmation orthogonal tests (FISH, NGS) for the detection of microsatellite instability and *NTRK* gene fusions.

**Figure 3:**
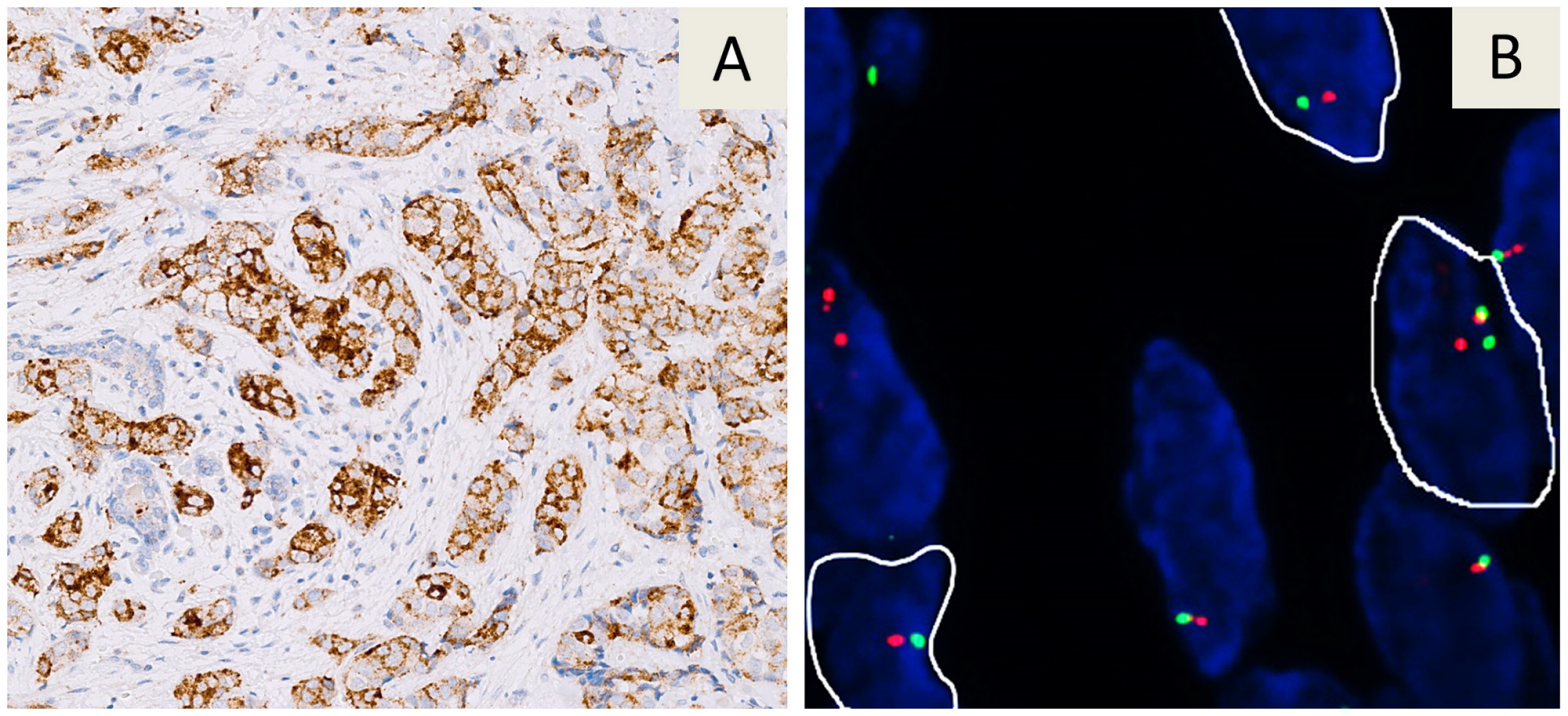
Immunohistochemical staining with the VENTANA pan-TRK (EPR17341) assay in a breast carcinoma A strong cytoplasmic immunoreaction is evident **(A)**. The tumor showed a *NTRK1* gene fusion by FISH analysis with a Break Apart probe. Nuclei highlighted with white borders show split signals **(B)**.

Large-scale analysis of MSI and *NTRK* fusions allowed to detect 48 (2.6%) MSI cases and 6 (0.3%) *NTRK* fusion cases in a series of 1865 malignant tumor samples examined, as reported in Table 2. In particular, IHC screening of mismatch repair proteins revealed an absence of nuclear staining within tumor cells in 53 (2.8%) cases of which 48 (91%) showed MSI by molecular analysis. The IHC screening test for TRK (A,B,C) proteins was found to be positive in 8 (0.4%) tumors. In 6 (75%) of these 8 tumors a *NTRK* gene fusion was shown by FISH. Two (0.3%) of 711 patients with ductal infiltrating breast carcinomas were found to be positive for *NTRK1* gene fusions (Figure 3) as well as a patient (0.8%) in a series of 132 cases with lung adenocarcinoma, 2 (1.3%) of 159 patients with gastrointestinal tumors, specifically a colorectal carcinoma and a gastrointestinal stromal tumor (GIST). In a series of 22 thyroid malignant tumors, a papillary thyroid carcinoma (4.5%) was found to be positive for a *NTRK3* fusion.

**Table 2:** Incidence of MMR deficiency, micro satellite instability and Neurothropic Receptor Tyrosine Kinase fusions in solid tumors

Malignant Tumor	Microsatellite Analysis				NTRK analysis		total
	dMMR	pMMR	MSI	MSS	Positive	Negative	
**Bladder**	1 (0.8)	128 (99.2)	1 (0.8)	128 (99.2)	0	129 (100)	**129**
**Prostate**	1 (0.6)	176 (99.4)	1 (0.6)	176 (99.4)	0	177 (100)	**177**
**Kidney**	1 (3.7)	26 (96.3)	1 (0.7)	26 (96.3)	0	27 (100)	**27**
**Testis**	0	9 (100)	0	9 (100)	0	9 (100)	**9**
**Penis**	0	3 (100)	0	3 (100)	0	3 (100)	**3**
**Gynecopathological**	12 (6)	189 (94)	10 (5)	191 (95)	0	201 (100)	**201**
**Gastrointestinal and hepatic**	23 (14.5)	136 (85.5)	21 (13.2)	138 (86.8)	2 (1.3)	157 (98.7)	**159**
**Pancreas**	1 (5.6)	17 (94.4)	1 (5.6)	17 (94.4)	0	18 (100)	**18**
**Lung**	1 (0.8)	131 (99.2)	1 (0.8)	131 (99.2)	1 (0.8)	131 (99.3)	**132**
**Thyroid**	0	22 (100)	0	22 (100)	1 (4.5)	21 (95.5)	**22**
**Salivary**	0	10 (100)	0	10 (100)	0	10 (100)	**10**
**Lymph Node**	0	78 (100)	0	78 (100)	0	78 (100)	**78**
**Oral cavity**	0	36 (100)	0	36 (100)	0	36 (100)	**36**
**Breast**	8 (1.1)	703 (98.9)	7 (1)	704 (99)	2 (0.3)	709 (99.7)	**711**
**Skin**	5 (3.3)	148 (96.7)	5 (3.3)	148 (96.7)	0	153 (100)	**153**
**Total**	53 (2.8)	1812 (97.2)	48 (2.6)	1817 (97.4)	6 (0.3)	1859 (99.7)	** 1865 (100)**

Abbreviations: dMMR, deficient Mismatch Repair; pMMR, proficient Mismatch Repair; MSI, Micro Satellite Instability; MSS, Micro Satellite Stable; NTRK, Neurothropic Receptor Tyrosine Kinase.

In 4 of these 6 cases, tissue was available for genomic assessment of the fusion patterns by NGS and/or multiplexing PCR/hybridization (Table 3). For two small samples included in the TSA series, NGS analysis could not be conducted due to the paucity of the starting material available. The TSA approach in these two cases has therefore allowed by IHC+FISH the identification of rare fusions otherwise undetectable by direct NGS analysis.

**Table 3:** TRK protein expression and NTRK gene fusions detected by immunohistochemistry, Fluorescence in situ hybridization and next generation sequencing in different tumor types

Tumor type	Histotype	NTRK Analysis		
		IHC	FISH	NGS
Gastrointestinal	Colorectal	cytoplasmic	NTRK1	NTRK1/TPM3
	GIST	cytoplasmic	NTRK1	NTRK1/MPRIP
Breast	Infiltrating ductal	cytoplasmic	NTRK1	NTRK1/LMNA
	Infiltrating ductal	perinuclear	NTRK1	NA
Lung	Adenocarcinoma	cytoplasmic	NTRK1	NTRK1/CD74
Thyroid	Papillary carcinoma	nuclear	NTRK3	NA

Abbreviations: GIST, gastrointestinal stromal tumor; IHC, immunohistochemistry; FISH, fluorescence in situ hybridization; NGS, next generation sequencing.

The diagnostic strategy described has enabled IHC analysis of up to 30 patients simultaneously per TMA and 6 patients simultaneously per TSA with a significant saving in terms of analytical costs and time. According to the equation reported in Figure 1B, it has been calculated that the analytical cost of IHC screening per TRK-positive patient on TMA samples was about 8 times the cost per single test and on TSA samples was about 42 times compared to 250 times if the IHC test had been carried on single samples. Using both TMA and TSA strategies an average 20 times saving was obtained.

**Figure 4:**
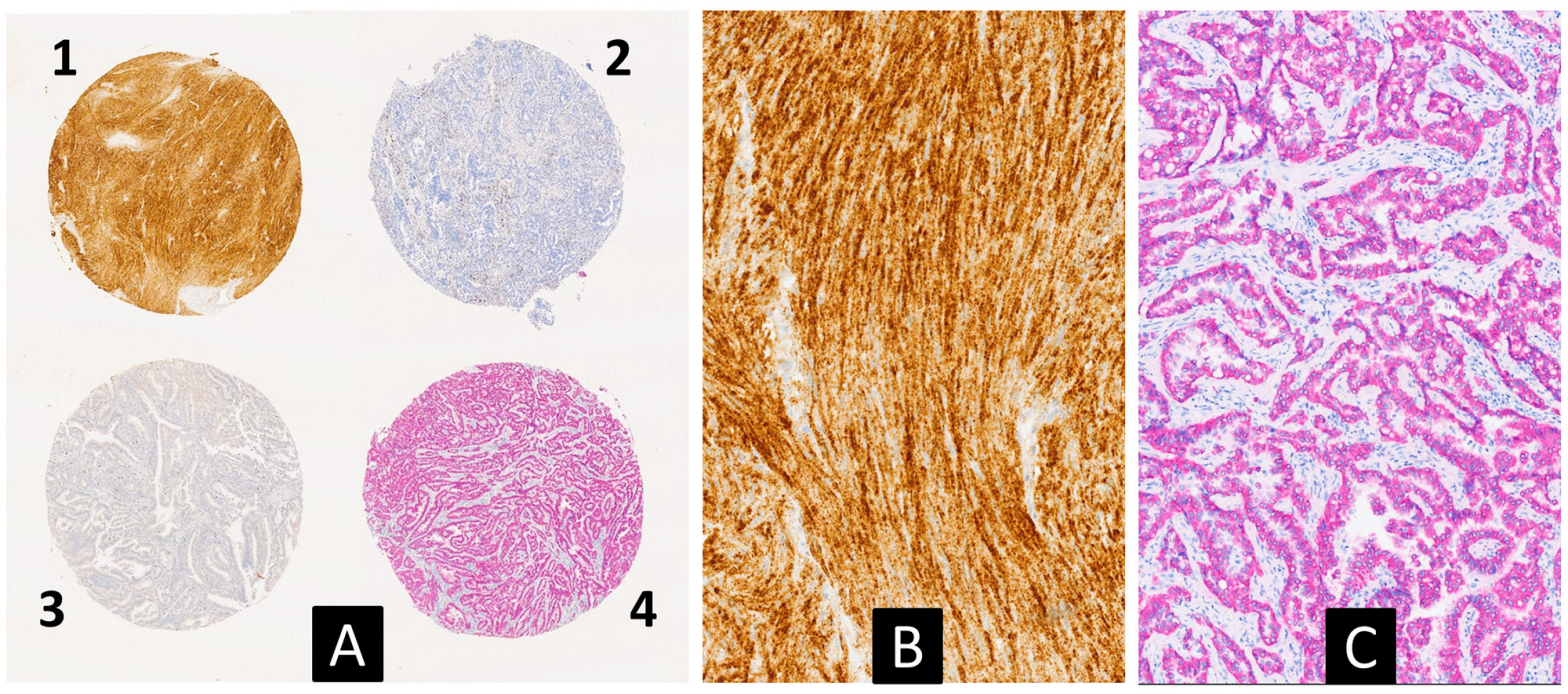
Multiplex immunohistochemical analysis for simultaneous detection of TRK (brown) and ALK1 (red) proteins on a multi-tumor TMA slide **(A)** (2.5×). Case 1 corresponds to a gastrointestinal stromal tumor, carring a *NTRK1* fusion, with a strong TRKA cytoplasmic expression (see also Table 3). Case 4 corresponds to a lung adenocarcinoma with an EML4-ALK fusion showing a cytoplasmic expression of the ALK1 protein. **(B** and **C)** are higher magnification pictures of cases 1 and 4, respectively (10×).

The high throughput of the described diagnostic strategy can be further enhanced by multiplex IHC on TMA sections/TSAs. In order to verify the feasibility of this approach, a section of a TMA with a positive TRKA sample and a positive ALK1 sample was subjected to multiplex IHC which simultaneously showed the different biomarkers in different colours (Figure 4).

## DISCUSSION

Among the driver mutations which can be targeted with specific treatments in different forms of human malignancies, there are uncommon genomic alterations affecting a minority of cancer patients [13, 14]. The assessment of rare but clinically important molecular alterations represents a challenge that, due to problems concerning diagnostic costs and time, can only be tackled efficiently with multiple simultaneous analyses. Two potential approaches are currently available in this regard. A first possibility is to analyze single samples for multiple alterations, this is possible through a series of multimarker techniques including massive parallel sequencing [15, 16], a second possibility is to simultaneously evaluate multiple samples with mono-(oligo-)marker screening technologies While the analysis of multiple alterations on a single sample does not alter a conventional prospective approach, the analysis of multiple samples per single marker requires a retrospective approach repeated prospectively at time intervals compatible with clinical needs. In the present study we have shown the feasibility of an innovative diagnostic strategy for the simultaneous analysis of rare mutations in multiple samples, based on the use of TMAs and TSAs. This has allowed to identify a series of patients affected by uncommon tumor types which can efficiently be treated with specific target therapies.

In particular, the method has been applied to the screening of patients affected by malignant tumors, irrespective of the histological types, for the detection of microsatellite instability and *NTRK* genetic fusions. Both of these two genetic alterations nowadays allow histology agnostic treatments [17].

It has been shown that metastatic or unresectable MSI-H patients or mismatch repair deficient (dMMR) metastatic or unresectable patients or dMMR patients affected by solid tumors that have progressed following previous therapies and who have no satisfactory alternative treatment options could be successfully treated with pembrolizumab. This has led the approval of pembrolizumab (Keytruda^®^, Merck & Co) by FDA in 2017, for a solid tumor treatment regardless the tumor primary site of origin and histology [18–20].

More recently, FDA and EMA have also approved the oral agent larotrectinib (VITRAKVI^®^, Loxo Oncology Inc. and Bayer) for treating metastatic or unresectable solid tumors of any type with a *NTRK* gene fusion. Moreover, FDA has granted a priority review designation to Entrectinib (RXDX-101, Roche Genentech) for patients with *NTRK* fusion–positive locally advanced or metastatic solid tumors [21–26].

Overall, in a series of 1865 cases of malignant tumors analyzed in this study during a trial period of 6 months, 48 cases with microsatellite instability in 9 different tumor forms and 6 cases of fusions in the *NTRK* gene family in 4 tumor forms were detected.

The number of cases identified does not yet allow a systematic statistical study of the incidence of these rare mutations in all types of solid tumors. This will be one of the aims of a multicenter study under the aegis of the Italian Society of Pathology (SIAPEC) that foresees the diffusion of this diagnostic approach to a series of reference centers of pathology in Italy that could allow to select patients on a large scale and to have rapid information on incidences. Theoretically, on the basis of the data obtained in our pilot study, considering at minimum to have available tissue in 60% of all cancer patients, we have estimated that a widespread diffusion of the strategy in Pathology Centers could allow us to identify more than 6.000 cases/year in Italy (26.000 cases/year in USA) with microsatellite alterations and more than 1000 cases/year in Italy (4500 cases/year in USA) with fusions of *NTRK* genes with consequent possible treatment of patients with effective drugs.

Other potential applications of this pathological strategy in oncology concern the IHC screening of several other genomic alterations including *ALK1* and *ROS1* fusions and *BRAF*-(V600E) mutations which may be drivers of various neoplastic forms, regardless of the tumor type, and could be treated in a near future with drugs already available for treatment of lung adenocarcinomas (*ALK1* and *ROS1*) and melanoma and lung carcinoma (*BRAF*) patients [2, 3, 27].

This diagnostic approach can potentially be applied to other clinical fields, even non-neoplastic, whenever it is necessary to identify a rare marker for which a rapid tissue screening method is available, such as IHC or FISH.

TMAs were first introduced in 1998, by J. Kononen and collaborators and since then used in research to study and validate cancer biomarkers in various patient cohorts [28–30]. We have generated and tested in a pathology department a diagnostic screening strategy based on the use of TMA samples. In addition we have developed a complementary diagnostic approach based on TSA which can be used to test multiple samples when the tissue in each case is very limited. By using both these approaches we could screen for rare mutations more than 95% of the tumors received in our Pathology Department. Based on this experience we have developed a new pathological workflow designed to be easily transferable in routine diagnostics, as reported in Table 4.

**Table 4:**
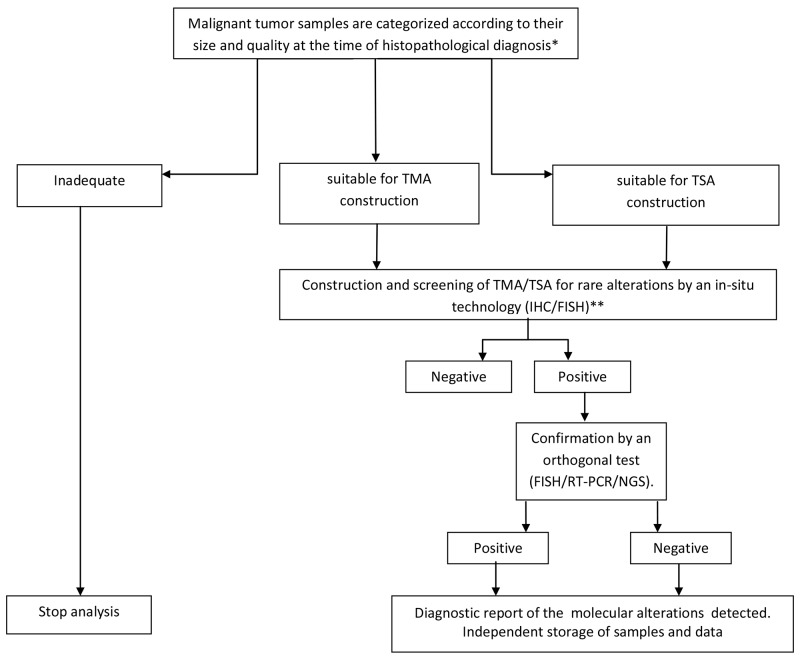
A pathological workflow for screening of rare genomic alterations on TMA/TSA samples in routine diagnostics ^*^Tissue categories are reported in the management software of the pathology department. At fifteen-days/1 month intervals (depending on the number of samples available) the recorded information are used for sample selection and construction of TMA and TSA, with the tissues arranged by organ pathology (See Material and Methods). ^**^In the case of neoplastic forms for which routine clinical molecular testing is planned (i.e non-small cell lung cancer, colorectal carcinoma, melanoma etc.), TMA and TSA construction must be performed after the analysis of conventional predictive tests. Abbreviations: IHC, immunohistochemistry; FISH, fluorescence in situ hybridization; RT-PCR, real-time PCR; NGS, next generation sequencing

The main limitation in the immediate application of this diagnostic strategy in all pathology departments are the limited diffusion of the TMA technology in diagnostic centers and the need of personnel with specific skills. However, the technology is not expensive and learning times are short. The specific steps that need to be implemented for this new activity can be easily addressed in most pathology departments. The selection of cases and areas of tissue at the time of reporting is conducted directly by the pathologist, the preparation of TMAs and IHC screening reactions at fortnightly intervals, require the work of a biomedical laboratory technician for about 5 days/month. Reading the IHC results on TMA/TSA samples by an experienced pathologist takes no more than 3 hours/month. TMA and TSA data can be managed by administrative staff within the management software of a pathological anatomy department, making minor changes to the software. We suggest an independent storage of TMAs at appropriate temperature (18–25°C) and controlled humidity. A diagnostic TMA bank within a pathological anatomy can have so many potential applications that the time and economic commitment necessary for its realization is in our opinion amply repaid. Based on this trial experience we have recently decided to adopt the TMA/TSA strategy in clinical practice. The approach was found to be easily adaptable to the routine workflow of our pathological department.

As reported in detail in the result section, the proposed diagnostic strategy can allow a substantial saving in terms of costs and time that makes feasible a diagnostic activity otherwise difficult to manage. The use of multiple IHC could further increase the throughput of the strategy. For example purposes only, we report for the first time, to the best of our knowledge, the possibility of simultaneously identifying different cancer biomarkers in different tumor forms (different cores) on the same TMA section. Considering the current progress of multiplexing technologies it is possible to hypothesize a multicolor analysis of several markers on the same TMA/TSA.

In the medium to long term future it can be hypothesized that multi-parametric molecular analyses such as massive parallel sequencing will be used for an accurate genomic characterization of all malignant tumors in order to treat patients with specific therapies [31]. An approach like the one presented here, essentially based on IHC screening of multiple samples may now be easily activated to implement these new therapeutic strategies. Furthermore, the described pathological approach has the advantage over genomic technologies of directly identifying the druggable targets, i.e the altered proteins, that can sometimes be the consequence of a post-transcriptional regulation which cannot be assessed by a genomic approach. In addition the TSA strategy described can allow the detection of rare alterations in cases with very limited amount of biologic material, insufficient for large scale genomic analysis. This could make the proposed diagnostic strategy a valid complement to massive parallel sequencing even when the latter method will eventually become the standard method of analysis, not to leave behind potential responders.

## MATERIALS AND METHODS

### Tissue samples

Tissue samples for this study were obtained from the archives of the Pathology Department - SS Annunziata Hospital, University of Chieti, the number based on the average volume for a six month period of routine activity. Table 1 shows the number of malignant tumors examined during a standard six month period in our Center, calculated on the average of the last five years. A numerically corresponding series of the different malignant tumors consecutively collected at our institution was used for this study. Informed consent was obtained from all patients and that the study was conducted in accordance with the precepts of the Helsinki Declaration.

Malignant tumor samples were subjected to histopathological revision and further processed in a dedicated workflow. Samples were categorized according to their size in “large” and “small”. Large samples were defined as those containing at least one fragment ≥ 0,5 cm^2^ of vital tumor tissue. From each large sample the most representative tumor areas were selected for TMA construction. All the other cases were indicated as small samples or, if very small, inadequate for additional processing.

### TMA and TSA construction

From each large sample, two (2mm) cores, captured in the selected areas, were deposited in a recipient 6×10 (60 cores) TMA block for simultaneous analysis of 30 patients, using the semi-automated Galileo CK3500 Micro Arrayer platform (Integrated Systems, Engineering srl, Milano). Internal specific control samples were added at the time of slide preparation.

For small samples, an innovative technique based on the preparation of Tissue Slice Array (TSA) was set up. The method consists of cutting 5 μm tissue slices from different original blocks and arranging up to 6 of them in an array on a single slide previously prepared with an alphabetized grid for patient identification (Figure 2).

### Immunohistochemical screening

TMA and TSA samples were screened by IHC for the expression of mismatch repair and TRK proteins in order to indirectly assess a potential MSI status or fusions in the *NTRK* gene family, respectively. The expression of mismatch repair proteins was evaluated on TSAs and 4 micron sections obtained from TMAs by IHC with anti-MLH1 (MutL Protein Homolog1, ES05 clone, Dako, Glostrup, Denmark), MSH2 (mutS protein homolog 2, FE11 clone, Dako, Glostrup, Denmark), PMS2 (post-meiotic segregation increate 2,EP51 clone, Dako, Glostrup, Denmark) and MSH6 (mutS protein homolog 6, EP49 clone, Dako, Glostrup, Denmark) antibodies on a DAKO Omnis platform, following the manufacturer’s protocol. Non neoplastic tissues, stromal cells and infiltrating lymphocytes were used as internal positive controls. Normal expression was defined as nuclear staining within tumor cells, while negative protein expression (suggesting a potential MSI) was defined as complete absence of nuclear staining within tumor cells with concurrent internal positive controls.

The expression of TRK proteins was assessed by the VENTANA pan-TRK (EPR17341) assay, based on a monoclonal primary antibody directed against the C-terminal region of the TRK proteins A, B and C, on the BenchMark XT Immunostainer platform, using the OptiView DAB IHC Detection Kit (Ventana, Tucson, Arizona, USA). A sample was considered as potentially rearranged if it showed a moderate/strong cytoplasmic, perinuclear and/or nuclear immunoreaction, as reported in literature. Sequential double staining of TRK and ALK1 proteins with two horseradish peroxidase chromogenic substrates (diaminobenzidine/Magenta), was performed using the pan-TRK (EPR17341) assay and the 5A4 clone (Novocastra, Leica Biosystems, Newcastle, Upon Tyne, UK) with the EnVision FLEX HRP Magenta, High pH (Dako Omnis).

### Confirmation by orthogonal methods

Cases found to be positive by IHC were tested with an orthogonal method. For the detection of MSI, fragment analysis by capillary gel electrophoresis was used. FISH, next generation sequencing (NGS) and multiplex PCR/hybridization were used for the detection of fusions in the *NTRK* gene family.

#### FISH analysis

FISH analysis was performed on unstained 4- to 5-micron, formalin-fixed, paraffin-embedded tumor tissue sections using a commercial *NTRK1-NTRK2-NTRK3* Break Apart FISH probe (Empire Genomics, Buffalo NY, USA) after pretreatment and denaturation steps, according to the manufacturers’ protocols.

FISH analysis was performed with a slide scanning system under a 60X oil immersion objective with a fluorescence microscope (Olympus BX61; Olympus Corporation, Tokyo, Japan). From 50 up to 150 cancer cells per patient were scored, and signals were evaluated using the FISH imaging and capturing software SoloTouch (Bioview Duet; BioView, Rehovat, Israel). Tumor samples were considered *NTRK* FISH positive if more than 15% of the tumor cells showed split red and green signals (signals separated by one or more signal diameters). Otherwise, the samples were considered FISH negative.

#### MSI analysis

After DNA extraction from FFPE samples, MSI testing was performed using the MSI Analysis System, Version 1.2 (Promega, Madison, WI, USA), a multiplex PCR assay that included primers fluorescently labeled for two pentanucleotide repeat markers, PentaC and PentaD and five mononucleotide repeat markers, NR21 (KIT), NR24 (ZNF2), BAT25 (SLC7A8), BAT26 (MSH2), and MONO27 (MAP4K3). After PCR, amplicons were detected by capillary electrophoresis on the ABI 310 Genetic Analyzer (Applied Biosystems, Foster City, CA, USA) and the results were analyzed using the GeneMapper software version 4.1 (Applied Biosystems). MSI status was defined as MSI-High, MSI-Low, or MS-Stable, depending on the number of mononucleotide markers with instability and corresponding to two or more (≥30%), one (<30% but >0%), or zero markers, respectively.

#### NGS analysis for NTRK rearrangements

TRK-positive cases were analyzed for NTRK1, 2 and 3 gene rearrangements by next generation sequencing using the Archer^®^ FusionPlex^®^ (ArcherDX, Boulder, CO, USA) panel on the Illumina Miseq System (Illumina, San Diego, Ca, USA). To this aim, RNA was extracted from formalin-fixed, paraffin-embedded (FFPE) tumor blocks by the QIAmp FFPE tissue kit (Qiagen, Antwerp, Belgium). After cDNA synthesis, a library of DNA fragments was constructed for targeted Anchored Multiplex PCR (AMP). AMPTM chemistry utilizes open-ended targeted amplification to identify gene fusions in a single sequencing assay, even without prior knowledge of fusion partners or breakpoints.

#### Multiplex PCR/hybridization

The IntelliplexTM RET/NTRK rearrangement test (PlexBio, Taipei, Taiwan) was used on a multiplexing technology based on two strategies, SelectAmp and πCode (PlexBio, Taipei, Taiwan). The first one uses the Locked Nucleic Acid (LNA) to block the PCR amplification of the wild-type sequence, dramatically increasing the sensitivity and the specificity. πCode strategy is based on MicroDisc, manufactured to generate up to 16,000 distinct circular image patterns for multiplexing applications. Each πCode has a distinct circular image pattern, which corresponds to a specific capture agent conjugated to the surface of the disc. All capture agent tagged πCode are pooled, enabling capturing and detection of specific analytes in one well reaction.
